# Red rice *koji* extract alleviates hyperglycemia by increasing glucose uptake and glucose transporter type 4 levels in skeletal muscle in two diabetic mouse models

**DOI:** 10.29219/fnr.v64.4226

**Published:** 2020-10-08

**Authors:** Takakazu Yagi, Koji Ataka, Kai-Chun Cheng, Hajime Suzuki, Keizaburo Ogata, Yumiko Yoshizaki, Kazunori Takamine, Ikuo Kato, Shouichi Miyawaki, Akio Inui, Akihiro Asakawa

**Affiliations:** 1 Department of Oral Health, Kobe-Tokiwa Junior College, Kobe, Japan; 2 Department of Orthodontics and Dentofacial Orthopedics, Kagoshima University Graduate School of Medical and Dental Sciences, Kagoshima, Japan; 3 Department of Pharmacological Sciences of Herbal Medicine, Kagoshima University Graduate School of Medical and Dental Sciences, Kagoshima, Japan; 4 Department of Medical Research, Chi-Mei Medical Center, Tainan City, Taiwan; 5 Department of Oral and Maxillofacial Surgery, Kagoshima University Graduate School of Medical and Dental Sciences, Kagoshima, Japan; 6 Kitasato University Hospital, Sagamihara, Japan; 7 Division of Shochu Fermentation Technology, Education and Research Center for Fermentation Studies, Faculty of Agriculture, Kagoshima University, Kagoshima, Japan; 8 Department of Medical Biochemistry, Kobe Pharmaceutical University, Kobe, Japan; 9 Department of Psychosomatic Internal Medicine, Kagoshima University Graduate School of Medical and Dental Sciences, Kagoshima, Japan

**Keywords:** red rice koji, streptozotocin, high fat diet, diabetes, GLUT4, plasma lipids

## Abstract

**Background:**

Red rice *koji* (RRK), prepared by growing *Monascus* species on steamed rice, has been reported to lower blood glucose levels in diabetic animal models. However, the action mechanism is not yet completely understood.

**Objective:**

The objective of this study was to examine the mechanism underlying the hypoglycemic action of RRK extract in two diabetic animal models: the insulin-deficiency mice, where the insulin deficiency was induced by streptozotocin (STZ), and insulin-resistance mice, where the insulin resistance was induced by a high-fat diet (HFD).

**Design:**

Low (12.5 mg/kg body weight [BW]) and high (50.0 mg/kg BW) doses of RRK extract were orally administered to the mice for 10 successive days (0.25 mL/day/mouse). The protein expression levels of glucose transporter type 4 (GLUT4) in the skeletal muscle and glucose transporter type 2 (GLUT2) in the liver were measured. Blood glucose (BG) levels of STZ-treated mice in insulin tolerance test (ITT) and BG and insulin levels of HFD-fed mice in intraperitoneal glucose tolerance test (IPGTT) were investigated.

**Results:**

In the STZ-treated mice, oral administration of RRK extract lowered BG levels and food intake but increased plasma 1,5-anhydroglucitol level. Moreover, the RRK extract lowered the BG levels of STZ-treated mice as measured by ITT. In the HFD-fed mice, we confirmed that the orally administered RRK extract lowered the BG and the homeostasis model assessment index for insulin resistance. Furthermore, the RRK extract lowered the BG and insulin levels of HFD-fed mice in IPGTT. Regarding the protein levels of GLUT, the orally administered RRK extract increased the GLUT4 level in the skeletal muscle; however, the RRK extract did not alter the GLUT2 level in the liver of either the STZ-treated or the HFD-fed mice.

**Discussion:**

Our study demonstrates that RRK extract can improve impaired glucose tolerance in mouse models of diabetes by enhancing GLUT4 expression in skeletal muscle.

**Conclusion:**

These results suggest that RRK extract could potentially be a functional food for the treatment of diabetes mellitus.

## Popular scientific summary

- Red rice *koji* (RRK) extract lowered blood glucose levels in streptozotocin (STZ)-treated and high-fat diet (HFD)-fed mice and food intake in STZ-treated mice.
- RRK extract lowered blood glucose levels of STZ-treated mice in insulin tolerance test and blood glucose and insulin levels of HFD-fed mice in intraperitoneal glucose tolerance test.
- RRK extract increased glucose transporter type 4 level in the skeletal muscle of both STZ-treated and HFD-fed mice.

In East Asia, natural food has consistently been used for medical cure and health improvement in humans, since long. The concept is based on the principle ‘Eating healthy prevents and cures disease’, followed in oriental medicine. Red rice *koji* (RRK), also called ‘beni *koji*’ in Japan and ‘hong qu’ in China, is one such a food that has been used not only as a source of fermented food and beverage but also as a traditional medicine across Asia, owing to its benefits to the digestive system and blood circulation system (1). RRK is produced by solid-state fermentation of *Monascus* species on steamed rice. It contains various substances, such as secondary metabolites, including monacolin K, and structural proteins present in mold, besides enzymes produced by the mold (2–4). Red *koji* has been reported to have anti-oxidative, antihypertensive, anti-hypercholesterolemic, antimicrobial, and anticarcinogenic effects in animal models and humans (1, 5–8). Shi and Pan had reported that oral administration of red *koji* mold lowers blood glucose (BG) levels and prevents pancreatic damage in rat models of streptozotocin (STZ)-induced diabetes (9, 10). STZ is well known to cause permanent loss of β cells and induce hyperglycemia and hypoinsulinemia (11). Recently, we have indicated that RRK powder reduces BG levels along with decrease in body weight (BW) and fat accumulation in high-fat diet (HFD)-fed obese mice. Although RRK extract was reported to increase the expression of glucose transporter type 4 (GLUT4) in a skeletal muscle cell line (12), the mechanisms of action involving the increase in GLUT4 in diabetic animal models are not understood completely.

Diabetes mellitus is associated with various diseases, including nephropathy, neuropathy, retinopathy, and cardiovascular disease (13–15), and has become a growing problem in both developed and developing countries around the world (16). Its etiology is related to high-calorie food consumption and unhealthy lifestyle practices. BG levels are physiologically controlled by insulin, which is secreted by β cells in the islets of Langerhans of pancreas. Dysfunction of insulin is the primary cause of diabetes; its deficiency in type 1 diabetes is induced by autoimmune destruction of β cells, and resistance to insulin results in type 2 diabetes, caused by obesity (17). Glucose transporters play a pivotal role in the regulation of BG levels. GLUT4 is a major glucose transporter that is primarily expressed in skeletal muscles and adipocytes, where it regulates cellular insulin-induced glucose uptake (18, 19). Glucose transporter type 2 (GLUT2) is expressed in the liver, where it modulates the passive movement of glucose across cell membranes (20). Therefore, it stands to reason that the expression of GLUT2 and 4 and their translocation into cell membranes are associated with the pathology of diabetes.

In this study, we investigated the effects of RRK extract on diabetes mellitus along with the underlying mechanisms, including GLUT4 in skeletal muscles and GLUT2 in liver, using two kinds of mouse models of diabetes: insulin-deficiency mice induced by an STZ and insulin-resistance mice induced by an HFD.

## Materials and methods

### Preparation of RRK extract

RRK extract was prepared as described in a previous study (21). In brief, rice (Japonica rice) was soaked in water for 1 h at 23 ± 1°C, and the excess liquid was drained off subsequently. The wet rice was steamed for 1 h at 100°C, and subsequently cooled to 40°C in a cabinet. The steamed rice was inoculated with red *koji* (*Monascus anka*), transferred to a sterile glass petri dish, and maintained at 35°C for 5 days. The RRK preparation was eventually freeze dried and ground to powder. RRK powder (500 g) was added to 1,500 mL of deionized water and allowed to stand overnight at 4°C. Next day, the mixture was centrifuged at 11,500×*g* for 15 min, and the supernatant was centrifuged at 21,500×*g* for 10 min. The final supernatant was used as an extract. This extract was concentrated in a rotary evaporator under reduced pressure at 60°C. The nutritional composition of RRK was based on the data obtained in a previous study (12), and no further details are available (Table 1).

**Table 1:** Nutritional composition of red rice koji (12)

Red rice *koji*	
Moisture (kcal/100 g fw)^a^	4.9
Protein (kcal/100 g fw)^a^	83.4
Carbohydrate (kcal/100 g fw)^a^	9.6
Fat (kcal/100 g fw)^a^	1.8
Ash (kcal/100 g fw)^a^	0.3
Glucose (kcal/100 g dw)^b^	15.0
Citric acid (kcal/100 g dw)^b^	104.0
Energy (kcal/100 g fw)^a^	388.0

a fw, fresh weight.

b dw, dry weight.

### Animals and experimental protocol

Six-week-old male C57BL/6 mice were obtained from Japan SLC, Inc. (Shizuoka, Japan) and the National Laboratory Animal Center (Tainan, Taiwan). All mice were kept in individual cages and maintained under controlled conditions (23 ± 1°C, 50 ± 10% humidity, and 12-h light–dark cycle with the light turned on at 7:00 A.M.) with free access to food and water. After acclimation for a week, the mice were used for experiments. During the experiments, food and water intake, and BW were recorded daily. All experimental procedures were performed according to the Japanese and Taiwanese national standardized guidelines. The experiments were approved by the Kagoshima University Committee for Animal Experiment (Institutional Review Board approval numbers MD 14085 and 14086) and the Department of Medical Research, Chi-Mei Medical Center (Institutional Animal Care and Use Committee approval number 105012242).

### Induction of diabetes in mice

In the mouse model of STZ-induced hyperglycemia, STZ (150 mg/kg BW; Sigma-Aldrich, St. Louis, MO, USA) was dissolved in a 10 mM sodium citrate buffer (pH 4.5) immediately before the intraperitoneal (IP) administration, as described in a previous study (21). BG levels were measured by a BG monitoring system (FreeStyle FLASHTM, Nipro, Osaka, Japan) 10 days after the administration of STZ. Mice were considered to be diabetic when the BG level was above 200 mg/dL in the overnight fasting state. In the HFD-fed mice, HFD (40% kcal fat from lipid, D08081001, Research Diets Inc., New Brunswick, Canada) was served for 4 successive weeks, and a standard diet was purchased from CLEA Japan, Inc. (CE-2, CLEA Japan, Tokyo, Japan). These mice were subsequently used in the study, during which HFD was served, as described previously (12).

### Treatment with RRK extract

Each group of diabetic mouse models was divided randomly into three subgroups as follows: (1) vehicle-treated group (distilled water [DW], 0.25 mL/mouse), (2) low dose of RRK extract (LD)-treated group (12.5 mg/kg BW), and (3) high dose of RRK extract (HD)-treated group (50 mg/kg BW). RRK extract was dissolved in DW and orally administered to the mice (0.25 mL/day/mouse) at 10:00 A.M. for 10 successive days. The mice for insulin tolerance test (ITT) or IP glucose tolerance test (IPGTT) were administered orally for 7 successive days before the test.

### Measurement of biochemical parameters and tissue isolation

Peripheral blood samples were collected into heparin tubes on day 10 from STZ-treated, HFD-fed, and vehicle-treated mice, fasting for 6 h under anesthesia, with IP administration of pentobarbital (150 mg/kg BW; Kyoritsu Seiyaku Corp., Tokyo, Japan). Mice were perfused with 0.1 M phosphate buffered saline (pH 7.0) to obtain liver and soleus muscle tissues, and the tissue samples were stored at −80°C until used for western blot analysis. The BG levels were measured by a BG monitoring system (FreeStyle FLASH^TM^, Nipro, Osaka, Japan). Plasma was separated by centrifugation at 4°C and stored at −80°C until assay. The levels of total cholesterol (T-CHO), high-density lipoprotein cholesterol (HDL-C), low-density lipoprotein cholesterol (LDL-C), and triglyceride (TG) in the blood samples were measured in Nagahama Biological Science Research Institute (Shiga, Japan). Insulin and 1,5-anhydroglucitol (1,5-AG) levels were determined using an ultra-sensitive mouse insulin ELISA (Morinaga Institute of Biological Science, Inc., Yokohama, Japan) and an enzyme-linked kit (Cloud-Clone Corp., Houston, TX, USA), respectively, according to the manufacturer’s instructions. Insulin resistance was measured using the homeostatic model assessment for insulin resistance (HOMA-IR). The HOMA-IR index was calculated according to the formula: fasting BG concentration (mmol/L) × fasting plasma insulin concentration (μU/mL)/22.5 (22).

### Insulin tolerance test and intraperitoneal glucose tolerance test (IPGTT)

Based on the measurement of different biochemical parameters, ITT was conducted for STZ-treated mice and IPGTT for HFD-fed mice at day 7 of RRK extract treatment. STZ-treated and HFD-fed mice were allowed to fast for 8 h before the test. In ITT, insulin (0.5 U/kg BW; Sigma-Aldrich, St. Louis, MO, USA) was administered intraperitoneally; peripheral blood samples were collected into heparin tubes from the tail vein at 0, 0.5, 1, 1.5, 2, 2.5, and 3 h after the IP administration of insulin. In IPGTT, glucose (1 mg/g BW; Sigma-Aldrich, St. Louis, MO, USA) was intraperitoneally administered. Peripheral blood samples were collected sequentially into heparin tubes from the tail vein at 0, 0.5, 1, 1.5, 2, and 2.5 h after the administration. Plasma was separated by centrifugation at 3,000×*g* for 15 min at 4°C, and stored at −80°C until assay. Areas under the curve (AUCs) for ITT and IPGTT were calculated using the Prism Software (GraphPad Software Inc., San Diego, CA, USA).

### Western blot analysis

Western blotting was performed to determine the GLUT4 level in skeletal muscle tissue (soleus muscle) and GLUT2 level in liver tissue. Approximately 40 mg of the soleus muscle and liver were homogenized separately in a 500 μL cold radio immune precipitation assay (RIPA) buffer (150 mM NaCl [Merck Millipore, Darmstadt, Germany], 50 mM Tris [Thermo Fisher Scientific Inc., Waltham, MA, USA], 5 mM ethylenediaminetetraacetic acid (EDTA), 50 mM NaF, 10 mM sodium pyrophosphate, 1 mM sodium orthovanadate [Thermo Fisher Scientific Inc., Waltham, MA, USA], 1% NP-40, 0.5% deoxycholate, 0.1% SDS [Merck KGaA, Darmstadt, Germany], pH 7.5 supplemented with 1 mM leupeptin, 1 μg/mL aprotinin, and 1 mM phenylmethylsulfonyl fluoride that was supplemented with protease and phosphatase inhibitor cocktail [Thermo Fisher Scientific Inc., Waltham, MA, USA]). The samples were sonicated for 20 sec and incubated at 4°C for 40 min. The insoluble material was removed by centrifugation (16,500×*g* for 15 min at 4°C). Protein concentration was determined by the bicinchoninic acid protein assay kit (Thermo Fisher Scientific Inc., Waltham, MA, USA). Samples (45 μL) were combined using 4 × reducing Laemmli sample buffer (240 mM Tris pH 6.8 [Thermo Fisher Scientific Inc., Waltham, MA, USA], 8% SDS, 40% glycerol, 50 mM EDTA, 1 M dithiothreitol (DTT) [Merck Millipore, Darmstadt, Germany], and 0.04% bromophenol blue [Bio-Rad Laboratories Inc., Hercules, CA, USA]) and then boiled at 95°C for 5 min. Thirty micrograms of protein sample was loaded per lane on a 10% sodium dodecyl-sulfate polyacrylamide gel electrophoresis (SDS-PAGE) gel, separated by electrophoresis, and transferred to a polyvinylidene fluoride (PVDF) membrane (Bio-Rad Laboratories Inc., Hercules, CA, USA). The membrane was incubated in 5% bovine serum albumin (Merck Millipore, Darmstadt, Germany) with Tris-buffered saline containing 0.1% Tween 20 (TBST) for 1 h at 25°C, washed with TBST thrice, and then probed with the following antibodies overnight at 4°C: anti-GLUT2 (1:1,000 dilution; ab54460, Abcam, Cambridge, UK), anti-GLUT4 (1:1,000 dilution; ab65267, Abcam, Cambridge, UK), and anti-β-actin (1:5,000 dilution; Merck Millipore, Darmstadt, Germany). After the primary antibody incubation, the membrane was washed thrice with TBST, and subsequently hybridized to horseradish peroxidase-conjugated anti-rabbit IgG antibody (Calbiochem, San Diego, CA, USA) or anti-mouse IgG antibody (1:5,000 dilution; Calbiochem, San Diego, CA, USA) for 1 h at 25°C. The immunoreactive bands were detected using an enhanced chemiluminescent (ECL) kit (Thermo Fisher Scientific Inc., Waltham, MA, USA) and by exposing an Hyperfilm ECL (GE Healthcare Life Sciences, Tokyo, Japan) to the membrane for 10 sec. Protein bands were captured on a CanoScan LiDE100 Scanner (Canon Inc., Tokyo, Japan). Optical densities of the bands were determined using Gel-Pro Analyzer Software 4.0 (Media Cybernetics, Silver Spring, MD, USA) and were normalized to beta-actin, used as a loading control on each blot.

### Statistical analysis

Statistical analyses were performed to determine the significant differences between multiple treatment groups, using one-way analyses of variance (ANOVA) followed by post hoc Tukey’s multiple comparison test, and two-way ANOVA followed by post hoc Sidak’s multiple comparison test. The tests were performed using Prism Version 6.0 Software (GraphPad Software Inc., San Diego, CA, USA). Results are presented as the means ± standard error of the mean (SEM); *P* values less than 0.05 are considered statistically significant.

## Results

### Effects of RRK extract on BW, food intake, water intake, blood glucose, 1,5-AG, HOMA-IR, and plasma lipids in STZ-administered mice

BW of mice in LD, HD, and vehicle groups, before the treatment, were 20.97 ± 0.42, 20.92 ± 0.38, and 20.66 ± 0.61 g, respectively, and there was no significant difference. For BW at day 10, there was no significant difference across the groups; however, the food intake of LD- and HD-treated mice was lower than that of vehicle-treated mice (*F*_2,18_ = 6.914, *P* = 0.0059, Table 2). There was no significant difference in water intake across the groups (Table 2). BG levels of LD- and HD-treated mice were significantly lower than those of vehicle-treated mice (*F*_2,15_ = 7.453, *P* = 0.0057, Table 2). Plasma 1,5-AG levels in LD- and HD-treated mice were higher than those in vehicle-treated mice (*F*_2,15_ = 7.453, *P* = 0.0057, Table 2). However, for insulin levels and HOMA-IR, there was no significant difference across the groups (Table 2), and also for plasma lipid levels (T-CHO, HDL-C, and LDL-C), there was no significant difference across the groups (Table 2).

**Table 2:** Values of body weight, food intake, water intake, blood glucose, 1,5-AG, insulin, and plasma lipids in STZ-treated mice with RRK extract treatment at day 10

	Vehicle	LD	HD
Body weight (g)	20.6 ± 0.8	20.3 ± 0.4	20.5 ± 0.5
Food intake (g)	5.8 ± 0.2	5.0 ± 0.2*	4.8 ± 0.3*
Water intake (g)	38.7 ± 1.0	39.6 ± 2.7	34.7 ± 1.4
Glucose (mg/dL)	499.0 ± 5.1	475.0 ± 6.4*	469.2 ± 5.9**
1,5-AG (μg/mL)	8.9 ± 0.6	17.2 ± 1.9*	26.3 ± 3.0^**,Φ^
Insulin (ng/mL)	0.3 ± 0.01	0.3 ± 0.02	0.3 ± 0.02
HOMA-IR	1.4 ± 0.05	1.3 ± 0.08	1.3 ± 0.08
T-CHO (mg/dL)	111.0 ± 9.0	111.4 ± 13.4	107.7 ± 11.8
HDL-C (mg/dL)	56.7 ± 3.2	56.3 ± 5.5	53.3 ± 5.9
LDL-C (mg/dL)	8.1 ± 1.1	8.9 ± 1.7	8.7 ± 1.2

LD, low dose of red rice *koji*; HD, high dose of red rice *koji*; 1,5-AG, 1,5-anhydroglucitol; HOMA-IR, homeostasis model assessment index for insulin resistance; T-CHO, total cholesterol; HDL-C, high-density lipoprotein cholesterol; LDL-C, low-density lipoprotein cholesterol.

Data are expressed as means ± standard error of the mean.

Statistical analyses were performed to determine the significant differences between multiple treatment groups, using one-way analyses of variance (ANOVA) followed by post hoc Tukey’s multiple comparison test.

* *P* < 0.05 vs vehicle

** *P* < 0.01 vs vehicle

Φ *P* < 0.05 vs LD.

### Effect of RRK extract on ITT

**Fig. 1:**
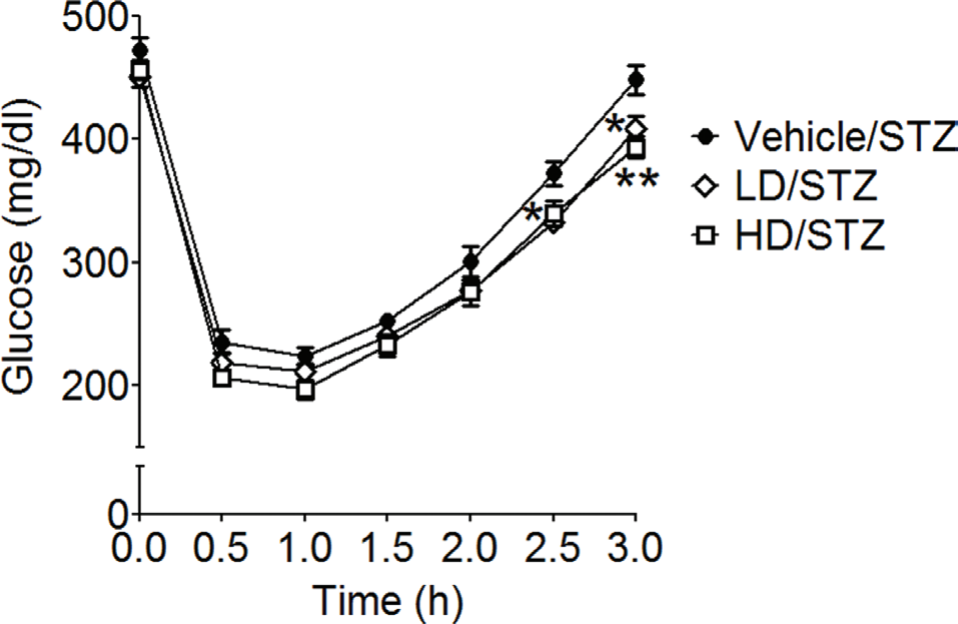
Red rice *koji* extract improved insulin sensitivity of mice with STZ-induced hyperglycemia. Data are expressed as means ± SEM (*n* = 6 each group). Statistical analyses were performed by two-way ANOVA followed by Bonferroni multiple comparisons. *P < 0.05 and **P < 0.01 were considered to be statistically significant when compared with vehicle-treated mice.

The BG levels of LD-treated mice at 2.5 and 3 h and of HD-treated mice at 3 h were significantly reduced than those of vehicle-treated mice (*F*_2,15_ = 5.07, *P* = 0.0208 in Fig. 1). The AUCs of BG levels in vehicle-, LD-, and HD-treated mice were 922.6 ± 23.7, 854.8 ± 12.8, and 838.2 ± 23.5, respectively, and the AUC of HD-treated mice was significantly lower than those of vehicle-treated mice (*F*_2,15_ = 4.762, *P* = 0.0250).

### Effects of RRK extract on BW, food intake, water intake, blood glucose, 1,5-AG, HOMA-IR, and plasma lipids in HFD-fed mice

BW of mice in LD, HD, and vehicle groups, before the treatment, were 25.3 ± 0.7, 27.3 ± 0.4, and 26.2 ± 0.4 g, respectively, and there was no significant difference. For BW, food intake, and water intake at day 10, there was no significant difference across the groups (Table 3). BG levels in LD- and HD-treated mice were significantly lower than those in vehicle-treated mice (*F*_2,15_ = 7.453, *P* = 0.0057, Table 3). In insulin levels, there was no significant difference across the groups; however, HOMA-IR in LD- and HD-treated mice was significantly lower than those in vehicle-treated mice (*F*_2,15_ = 15.53, *P* = 0.0002, Table 3). The plasma levels of T-CHO, HDL-C, LDL-C, and TG in LD- and HD-treated mice were significantly lower than those in vehicle-treated mice (*F*_2,16_ = 17.30, *P* < 0.0001; *F*_2,16_ = 10.53, *P* = 0.0012; *F*_2,16_ = 12.67, *P* = 0.0005; *F*_2,15_ = 6.738, *P* = 0.0082; respectively, in Table 3).

**Table 3:** Values of body weight, food intake, water intake, blood glucose, 1,5-AG, insulin, and plasma lipids in HFD-fed mice with RRK extract treatment at day 10

	Vehicle	LD	HD
Body weight (g)	25.7 ± 0.5	26.4 ± 0.5	25.0 ± 0.3
Food intake (g)	2.6 ± 0.2	2.2 ± 0.04	2.2 ± 0.08
Water intake (g)	3.7 ± 0.5	3.5 ± 0.2	4.1 ± 0.5
Glucose (mg/dL)	151.7 ± 3.3	138.2 ± 2.3**	125.7 ± 2.0^**,Φ^
Insulin (ng/mL)	2.2 ± 0.06	2.1 ± 0.06	2.1 ± 0.05
HOMA-IR	3.1 ± 0.04	2.7 ± 0.07**	2.5 ± 0.04**
T-CHO (mg/dL)	91.3 ± 5.1	72.7 ± 1.7**	65.5 ± 1.9**
HDL-C (mg/dL)	40.5 ± 2.3	34.6 ± 0.9**	30.5 ± 1.2**
LDL-C (mg/dL)	46.0 ± 3.4	34.9 ± 1.0**	31.9 ± 1.0**
TG (mg/dL)	27.4 ± 4.6	16.0 ± 1.2*	15.3 ± 1.6*

LD, low dose of red rice *koji*; HD, high dose of red rice *koji*; 1,5-AG, 1,5-anhydroglucitol; HOMA-IR, homeostasis model assessment index for insulin resistance; T-CHO, total cholesterol; HDL-C, high-density lipoprotein cholesterol; LDL-C, low-density lipoprotein cholesterol; TG, triglyceride.

Data are expressed as means ± standard error of the mean.

Statistical analyses were performed to determine the significant differences between multiple treatment groups, using one-way analyses of variance (ANOVA) followed by post hoc Tukey’s multiple comparison test.

* *P* < 0.05 vs vehicle

** *P* < 0.01 vs vehicle

Φ *P* < 0.05 vs LD.

### Effect of RRK extract on IPGTT

**Fig. 2:**
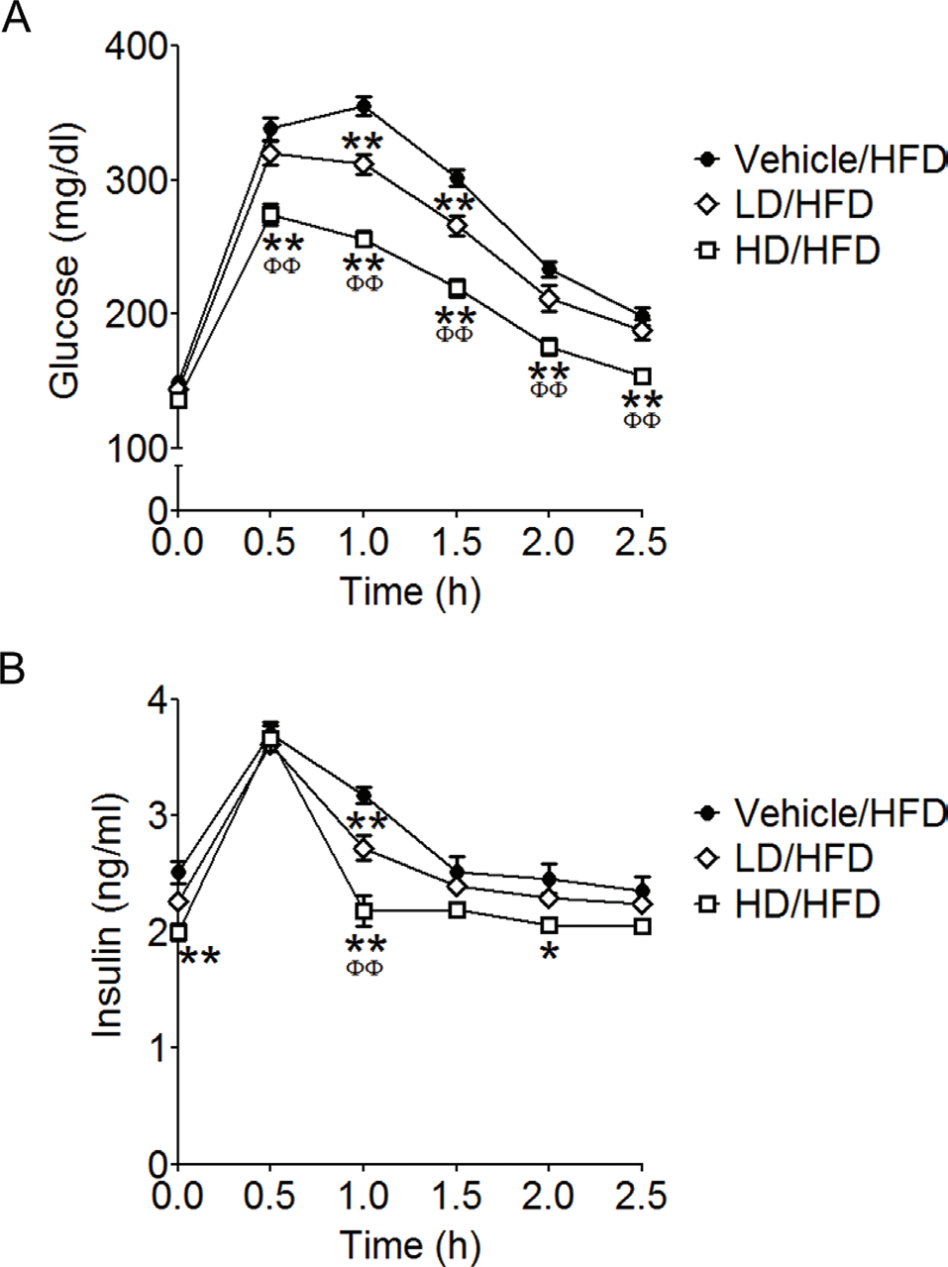
Red rice *koji* extract improved glucose tolerance in HFD-fed mice. Blood glucose levels (A) and plasma insulin levels (B) in glucose tolerance test. Data are expressed as means ± SEM (*n* = 6 each group). Statistical analyses were performed by two-way ANOVA, followed by Bonferroni multiple comparisons. *P < 0.05 and **P < 0.01 were considered to be statistically significant compared with vehicle-treated mice. ^ΦΦ^*P* < 0.01 was considered to be statistically significant when compared with LD-treated mice.

The BG levels of LD-treated mice at 1 and 1.5 h and of HD-treated mice at 0.5–2.5 h were significantly reduced than those of vehicle-treated mice (*F*_2,15_ = 29.86, *P* < 0.0001 in Fig. 2A). The AUCs of vehicle-, LD-, and HD-treated mice were 700.9 ± 13.4, 637.6 ± 17.5, and 534.8 ± 13.0, respectively; the AUCs of BG levels in HD- and LD-treated mice were significantly lower than those in vehicle-treated mice, and in HD-treated mice were significantly lower than those of LD-treated mice (*F*_2,15_ = 32.14, *P* < 0.0001). Plasma insulin levels of LD-treated mice at 1 h and of HD-treated mice at 0, 1, and 2 h were significantly lowered as compared with those in vehicle-treated mice (*F*_2,15_ = 8.33, *P* = 0.0037 in Fig. 2B). The AUCs of plasma insulin levels in vehicle-, LD-, and HD-treated mice were 7.2 ± 0.3, 6.6 ± 0.08, and 6.1 ± 0.2, respectively, and the AUC of HD-treated mice was significantly lower than those of vehicle-treated mice (*F*_2,15_ = 8.526, *P* = 0.0034).

### Effects of RRK on the expression of GLUT4 in skeletal muscle and GLUT2 in liver

**Fig. 3:**
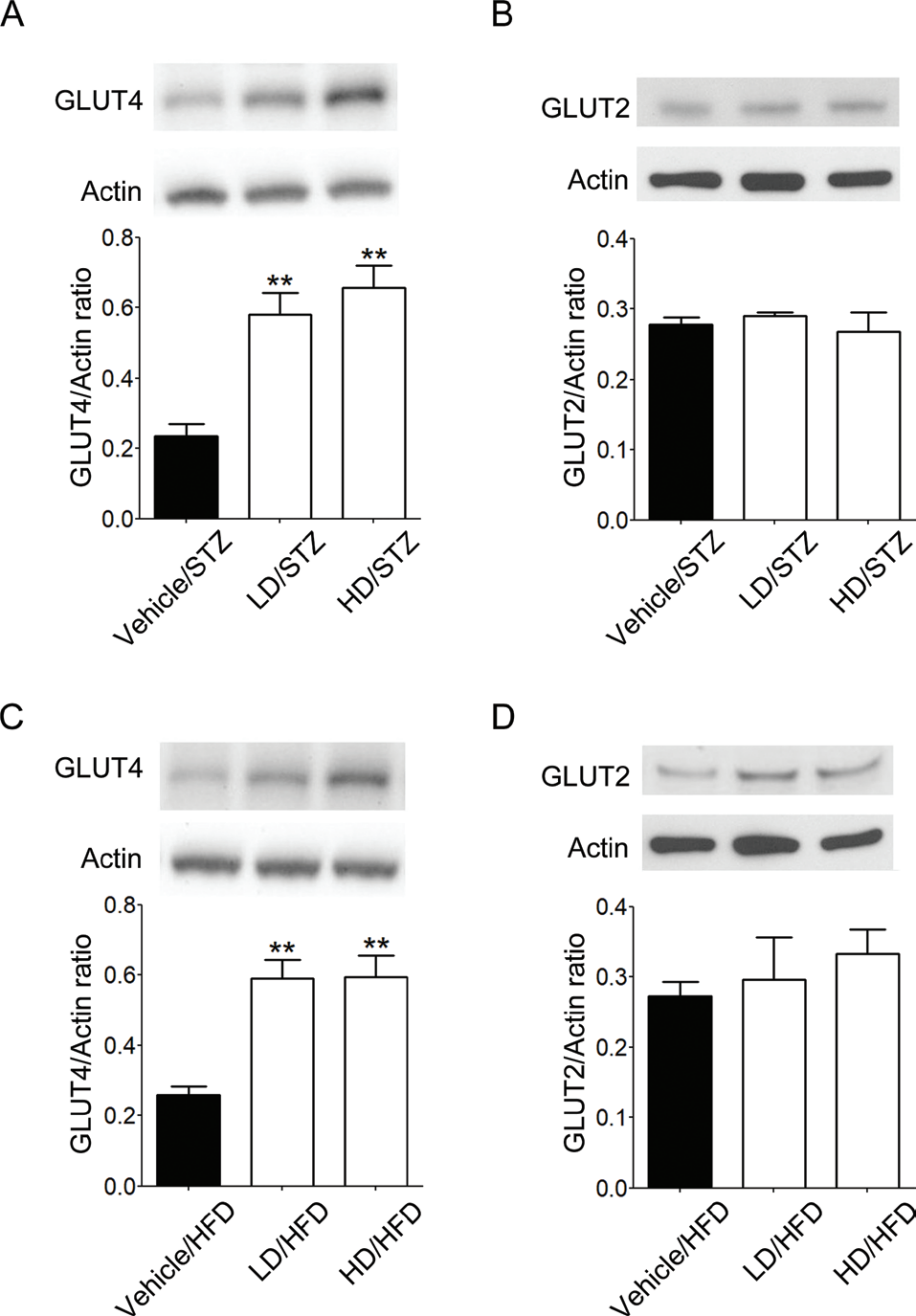
Red rice *koji* extract enhanced GLUT4 protein level in skeletal muscle, although it had no effect on GLUT2 protein level in the liver of STZ-treated and HFD-fed mice. Expressions of GLUT4 and GLUT2 in STZ-treated (A, B) and HFD-fed mice (C, D) were measured by Western blot analysis. Representative photographs of Western blot analysis were shown. Data are represented as means ± SEM (*n* = 4 each group). Statistical analysis was performed by one-way ANOVA followed by Tukey’s test. **P < 0.01 compared with vehicle-treated mice.

Expression of GLUT4 was significantly increased by the administration of RRK extract in muscle tissues of both STZ-treated and HFD-fed mice (*F*_2,9_ = 15.67, *P* = 0.0012 in Fig. 3A; *F*_2,9_ = 16.55, *P* = 0.0010 in Fig. 3C). However, there was no difference in the expression of GLUT2 in liver tissues of both STZ-treated and HFD-fed mice (Fig. 3B, D).

## Discussion

Fermented products, such as cheese, bread, and yogurt, are known to contribute to the promotion of health, as functional foods. Red *koji* is one of such fermented food, which is prevalent in Asia, especially, Japan, China, and Korea. RRK includes several bioactive metabolites: isoflavones, dimerumic acid, polyketide monacolins, monascin, and γ-amino acids (23). Isoflavones and these glycosides have been reported to produce antioxidant effect (24) besides lowering LDL-C (25) levels. Monacolin K, one of the ingredients in RRK, has been reported to inhibit inflammation, improve insulin resistance, and reduce plasma lipid levels (26, 27).

In the present study, we investigated hypoglycemic and hypolipidemic effects of two dosages of RRK extract in STZ-administered and HFD-fed mice. Oral treatment with RRK extract for 10 days was able to lower the BG levels in both STZ-treated and HFD-fed mice; the 1,5-AG is resorbed through fructose and mannose active transporters, and the balance of serum concentration is maintained by other transporters. In hyperglycemia, renal glucose level is higher than the resorption capacity of glucose transporters; thus, glucose resorption occurs via the fructose and mannose transporters and leads to the inhibition of reabsorption of 1,5-AG in renal tubules. Therefore, 1,5-AG level decreases in patients with diabetes and has thus been used as a marker of short-term glycemic control (28), as an alternative to the current glycemic markers such as hemoglobin A1c (29, 30). In our study, orally administered RRK extract remarkably increased the plasma concentrations of 1,5-AG in STZ-treated mice in a dose-dependent manner. In the HFD-fed group, RRK extract treatment lowered the plasma levels of T-CHO and LDL remarkably. More favorable hypolipidemic effects were observed in the HD group as compared with those in the LD group. These results indicated that RRK extract has a beneficial effect on glucose metabolism in diabetic mice.

Diabetes mellitus is the most common endogenous cause of fat metabolism-disorder (31). In several large prospective clinical trials, red *koji* was found to be useful in the primary and secondary prevention of heart disease and other complications of atherosclerosis (32, 33). In the HFD-fed group, RRK extract treatment remarkably lowered the plasma levels of T-CHO and LDL. More favorable hypolipidemic effects were observed in the HD group than in the LD group. STZ is known to induce hyperphagia in mice (34). In our study, consistent with a previous report (35), RRK extract treatment reduced food intake in STZ-treated diabetic mice. These results indicated that RRK extract has therapeutic efficacy in ameliorating hyperphagia in addition to lowering BG and lipid levels. Although RRK extract did not alter the plasma insulin levels, results from the IPGTT and ITT showed that RRK extract could improve glucose utilization and insulin sensitivity in HFD-fed and STZ-treated diabetic mice. In addition, RRK extract decreased HOMA-IR index in the HFD-fed mice.

Skeletal muscle is the predominant tissue for insulin-stimulated glucose uptake and GLUT4 (36). GLUT4 is an insulin-responsive glucose transporter that mediates glucose influx in the muscle cells. It has been reported that GLUT4 expression and translocation could be regulated through the G-protein-phospholipase C-protein kinase C and 5’ adenosine monophosphate (AMP)-activated protein kinase pathway in muscle cells (37). Impaired expression of GLUT4 has been reported to be linked to obesity, type 1 diabetes, and type 2 diabetes (38). In the present study, treatment with RRK extract increased skeletal muscle GLUT4 levels in the STZ-treated type 1 diabetic mice and the HFD-fed type 2 diabetic mice. These results were consistent with those of our *in vitro* study, wherein the RRK extract upregulated GLUT4 expression levels in L6 myotube cells (12). In this study, there was no significant difference in the expression of GLUT2 in the liver after treatment with the RRK extract in either of the diabetic models, thereby suggesting that the RRK extract may regulate glucose metabolism primarily through the regulation of GLUT4 in skeletal muscles. However, there are a few limitations of the present study. Additional parameters will be included in the future studies to investigate the role of the RRK extract in diabetes, such as investigation of changes in adipocytes size, energy utilization aspects, signaling pathways for energy utilization, and changes in inflammatory adipokine levels. Moreover, the GLUT4 translocation assay also needs to be performed.

It was recently reported that metabolites produced by intestinal bacteria regulate β-cell functions in diabetes (39). In addition, foods fermented with *koji* mold activate intestinal bacteria (40). Therefore, fermented foods may contribute to the improvement of diabetes through the activation of intestinal bacteria.

## Conclusion

The RRK extract showed the hypoglycemic effect in STZ-treated and HFD-fed mice via promoted GLUT4 expression in skeletal muscle. It is suggested the possibility of RRK extract being used as a novel hypoglycemic agent for the treatment of diabetes.
